# Atomistic Mechanisms of Silicone Rubber Degradation Under Coupled Temperature–Humidity–Electric Field Conditions

**DOI:** 10.3390/polym18121530

**Published:** 2026-06-19

**Authors:** Yiheng Zhou, Zhijun An, Yixin He, Cong Qian, Qiuhua Zhou, Wentian Zeng, Xinhan Qiao, Wenyu Ye

**Affiliations:** 1 School of Information and Control Engineering, School of Electrical Engineering, China University of Mining and Technology, Xuzhou 221116, China; zhouyiheng55@163.com (Y.Z.); ts24230001a31@cumt.edu.cn (Z.A.); ts24230090p31@cumt.edu.cn (Y.H.); ts23230005a31@cumt.edu.cn (W.Z.); ywypost@cumt.edu.cn (W.Y.); 2 State Grid Jiangsu Electric Power Co., Ltd., Nantong Power Supply Branch, Nantong 226007, China; qiancong_sgcc@163.com (C.Q.); zhouqiuhua_sgcc@163.com (Q.Z.)

**Keywords:** silicone rubber, reactive molecular dynamics, ReaxFF, temperature–humidity–electric field coupling, Si–O bond cleavage, degradation mechanism

## Abstract

Silicone rubber is an important external insulating material for composite bushings, composite insulators, and other power equipment. During long-term service, it is inevitably exposed to coupled environmental and electrical stresses, such as elevated temperature, moisture ingress, strong electric fields, and partial discharge, which may lead to hydrophobicity loss, surface chalking, crack propagation, and particle shedding. To reveal the microscopic degradation mechanism of silicone rubber under complex operating conditions, a molecular model of methyl vinyl silicone rubber was constructed using Materials Studio. A stable silicone rubber molecular structure was obtained through crosslinking, geometry optimization, and ensemble relaxation. Subsequently, a reactive molecular dynamics simulation system under coupled temperature–humidity–electric field conditions was established using LAMMPS and the ReaxFF reactive force field. Different temperature gradients, electric field intensities, and aging–recovery stages were designed to investigate the degradation behavior of silicone rubber. The evolution of the maximum carbon content, maximum silicon content, carbon-containing decomposition products, and typical small-molecule products, including H_2_, H_2_O, CH_4_, C_2_H_2_, C_2_H_4_, and C_2_H_6_, was statistically analyzed. In addition, atomic trajectory tracking was performed to clarify the processes of methyl group detachment, Si-O bond cleavage, water molecule participation, and molecular chain reconstruction. The results show that high temperature mainly promotes methyl group detachment from side chains and fracture of the siloxane main chain, while a strong electric field accelerates the decomposition process and induces the transformation of long siloxane chains into shorter chains. Water molecules can react with broken siloxane chains to form hydroxyl-containing structures, making the structural degradation partially irreversible. The degradation process of silicone rubber under coupled temperature–humidity–electric field stress can be summarized as side-chain detachment, main-chain scission, water-assisted reactions, free-radical recombination, and local molecular aggregation. This study provides a molecular-level theoretical basis for aging mechanism analysis, condition assessment, and lifetime prediction of composite external insulating materials.

## 1. Introduction

Silicone rubber has been widely used in composite bushings, composite insulators, cable accessories, and other high-voltage external insulation systems because of its excellent hydrophobicity, pollution flashover resistance, weather resistance, and electrical insulation properties [1,2,3]. In practical service environments, silicone rubber external insulation is continuously exposed to complex environmental and electrical stresses, including elevated temperature [4], moisture ingress [5], ultraviolet radiation [6], strong electric fields [7], corona discharge [8], and surface arc discharge [9]. With increasing service time, the material surface may gradually suffer from hydrophobicity loss, hardening, chalking, cracking, and particle shedding [10]. These degradation phenomena weaken the mechanical integrity and electrical reliability of the external insulation system and may eventually lead to partial discharge, surface flashover, or even insulation failure [11]. Recent studies have also shown that damp-heat aging and multi-factor aging can significantly affect the structural evolution, hydrophobicity, and macroscopic properties of silicone rubber insulation materials [12]. Therefore, revealing the degradation mechanism of silicone rubber under coupled multi-stress conditions is of great significance for condition assessment, lifetime prediction, and reliable operation of composite external insulation equipment.

Previous studies on silicone rubber aging can be broadly divided into several categories. Experimental aging studies have used scanning electron microscopy, Fourier-transform infrared spectroscopy, thermogravimetric analysis, hydrophobicity tests, and dielectric measurements to characterize surface morphology, chemical functional groups, thermal stability, and electrical properties after aging [13,14]. Conventional molecular dynamics studies have provided information on chain mobility, diffusion behavior, and structural relaxation of polymeric insulation materials [15]. In contrast, ReaxFF reactive molecular dynamics can describe bond cleavage, bond formation, and free-radical reactions, and is therefore more suitable for analyzing the degradation pathways of siloxane polymers [16,17,18]. However, studies that simultaneously consider temperature, humidity, and electric field stresses at the atomic scale remain limited. This limitation motivates the present work.

Reactive molecular dynamics simulations provide an effective approach for describing bond breaking and formation at the atomic scale, and it is particularly suitable for investigating the decomposition pathways and product evolution of polymer materials under high-temperature and electric-field environments [19,20]. The ReaxFF reactive force field can dynamically simulate chemical bond cleavage, recombination, and free-radical reactions in molecular systems, providing a powerful tool for studying the aging mechanism of silicone rubber. Molecular simulation has also been applied to other polymeric insulation systems, such as cross-linked polyethylene [21,22], to reveal the relationship between structural evolution, crosslinking modes, thermal-oxidative aging, mechanical properties, and insulation performance [23,24]. By combining molecular dynamics simulations with product statistics and trajectory analysis, the microscopic processes of side-chain detachment, main-chain fracture, water-assisted reactions, and decomposition product formation during silicone rubber degradation can be systematically analyzed [25,26].

Current molecular dynamics (MD) investigations into silicone rubber (SIR) aging predominantly focus on elucidating the pyrolysis mechanisms induced by individual environmental factors, such as temperature [13], humidity [27], and electric fields [28]. However, in practical operating conditions, insulation equipment is typically subjected to complex, coupled multi-factor stresses, wherein distinct aging factors exert synergistic or interactive effects [29,30]. Currently, there remains a notable deficiency in microscopic research regarding the degradation mechanisms under such coupled conditions. Therefore, to further elucidate the degradation behaviors of SIR under realistic service environments, it is imperative to investigate the pyrolysis mechanisms of SIR under the synergistic coupling of multiple aging factors.

Although previous studies have provided important information on the macroscopic aging behavior of silicone rubber, several microscopic issues remain unresolved [31]. First, most experimental studies describe changes in surface morphology, hydrophobicity, functional groups, and thermal stability, but they cannot directly identify the sequence of bond breaking, free-radical reactions, and molecular reconstruction. Second, existing molecular simulations of silicone rubber or PDMS mainly focus on thermal decomposition or single-stress aging, while the coupled effects of temperature, moisture, and electric field are less frequently considered. Third, the role of water molecules in Si-O backbone cleavage and hydroxyl-containing structure formation has not been sufficiently clarified at the atomic scale. Therefore, the specific advance of this work is to establish a ReaxFF reactive molecular dynamics model of methyl vinyl silicone rubber and reveal the degradation pathway involving side-chain detachment, Si-O main-chain scission, water-assisted reactions, free-radical recombination, and local molecular aggregation under coupled temperature–humidity–electric field conditions [32].

In this study, methyl vinyl silicone rubber is selected as the research object. A silicone rubber molecular model is first constructed using Materials Studio, and a stable crosslinked structure is obtained through crosslinking, geometry optimization, and ensemble relaxation. Water molecules are then introduced into the model to represent a humid environment. Based on LAMMPS and the ReaxFF reactive force field, reactive molecular dynamics simulations are carried out under coupled temperature–humidity–electric field conditions with different temperature gradients and electric field intensities.

The evolution of the maximum carbon content, maximum silicon content, carbon-containing products, and typical small-molecule products during thermal decomposition under different electric fields is analyzed. The decomposition behavior of siloxane chains under an electric field alone and the molecular reconstruction behavior during the recovery stage are further investigated. Moreover, the role of water molecules in accelerating irreversible degradation is clarified through atomic trajectory analysis. Finally, the microscopic degradation pathway of silicone rubber under coupled temperature–humidity–electric field stress is summarized from the perspectives of side-chain cleavage, main-chain scission, free-radical recombination, and molecular aggregation. The results are expected to provide theoretical support for aging diagnosis and operational reliability assessment of composite external insulating materials. Compared with previous studies mainly focusing on thermal decomposition or hygrothermal aging, this work further considers the coupled temperature–humidity–electric field environment and clarifies the water-assisted Si-O bond cleavage and reconstruction pathway at the atomic scale.

## 2. Siloxane Model Construction and Simulation Conditions

### 2.1. Simulation Software Platform

The simulation software used in this study includes Materials Studio 2020, developed by BIOVIA [33,34], and LAMMPS 2020, developed by Sandia National Laboratories. Specifically, Materials Studio was employed to construct the siloxane models and track the subsequent reaction trajectories. Meanwhile, LAMMPS was utilized to perform coupled temperature–humidity–electric field molecular dynamics (MD) simulations on the models, thereby revealing the pyrolysis mechanisms of the aged siloxane.

### 2.2. Construction of Silicone Rubber Molecular Model

**Figure 1:**
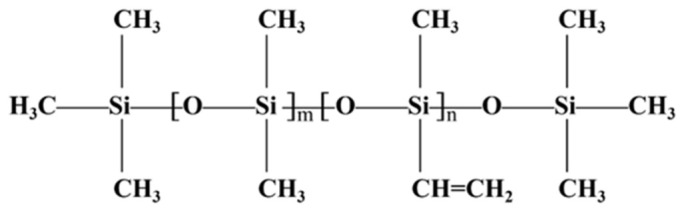
Molecular structure of MVQ.

Silicone rubber is mainly composed of methyl vinyl silicone rubber (MVQ), and its chemical structure is shown in Figure 1, where m is much larger than n. For silicone rubber materials used in composite bushings, the vinyl content is generally in the range of 0.03–2.1%.

**Figure 2:**
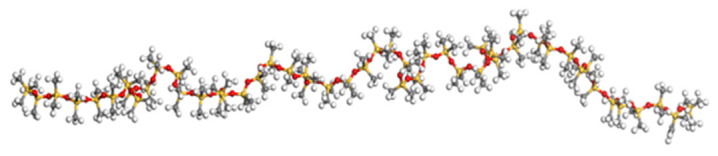
Optimized siloxane chain model.

Accordingly, a methyl vinyl silicone rubber model with a degree of polymerization of 50 was constructed. To balance computational efficiency and model validity, m and n were set to 48 and 1, respectively, which corresponds to a vinyl content of 1.84%. The constructed MVQ chain contained 105 carbon atoms, 50 oxygen atoms, 51 silicon atoms, and 312 hydrogen atoms, with the vinyl group located at the end of the repeating unit. Due to the constraints on vinyl content, increasing the number of vinyl groups would double both the overall size of the model and the computational cost. Therefore, considering model rationality and computational efficiency, the current model was adopted in this study. The single-chain silicone rubber molecule was subjected to energy minimization and geometry optimization using Materials Studio, and a reasonable geometric configuration was obtained, as shown in Figure 2. In the figure, yellow represents silicon atoms, red represents oxygen atoms, black represents carbon atoms, and white represents hydrogen atoms.

**Figure 3:**
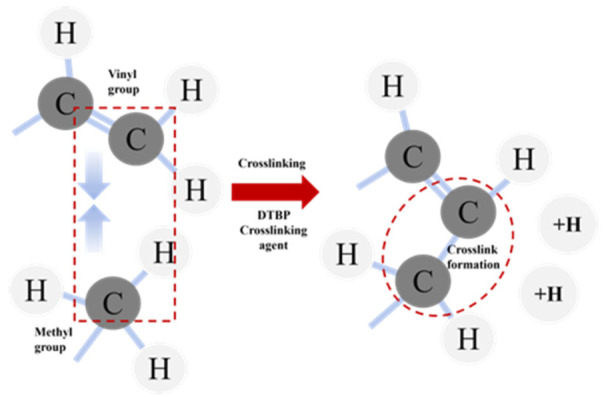
Schematic of the siloxane crosslinking reaction.

An amorphous cell containing four MVQ chains was then constructed using the Amorphous Cell module, and the sample was crosslinked using a Perl script. Crosslinking refers to the formation of chemical bonds between polymer chains, transforming them into a complex network structure. A common crosslinking reaction of MVQ involves bond formation between vinyl groups and methyl groups under the action of a crosslinking agent. The schematic diagram of the siloxane crosslinking reaction is shown in Figure 3. This study simulates the crosslinking reaction of silicone rubber chains mediated by the DTBP crosslinker. Since DTBP does not persist in the final crosslinked product and functions in a manner analogous to a catalyst, the direct incorporation of the crosslinker was omitted to simplify the simulation workflow. Instead, a Perl script was employed to computationally mimic the crosslinking effects of DTBP.

The crosslinking procedure for the silicone rubber model was initiated by identifying the reactive sites on both the silicone rubber chains and the crosslinking agent. Subsequently, the silicone rubber model was cross-linked using a Perl script. The final cross-linking degree was set to 4%, with an initial reaction radius of 3 Å, a maximum reaction radius of 8 Å, and 3 iterations. The system was then subjected to geometry optimization and molecular dynamics relaxation to obtain a stable molecular configuration. During each iteration, the algorithm evaluated whether the crosslinking criteria were satisfied within the current reaction radius. When the criteria were met, the crosslinking reaction was performed, followed by further geometry optimization and relaxation. If the current reaction radius exceeded the predefined maximum value, the iteration counter was reset, and the reaction radius was increased to provide additional opportunities for crosslink formation. This iterative process was continued until the target crosslinking degree was achieved or the maximum number of iterations was reached. Upon completion, the crosslinking information was recorded, and the final crosslinked model was exported. This procedure ensured that the silicone rubber network reached the required crosslinking density while maintaining structural stability throughout the simulation.

**Figure 4:**
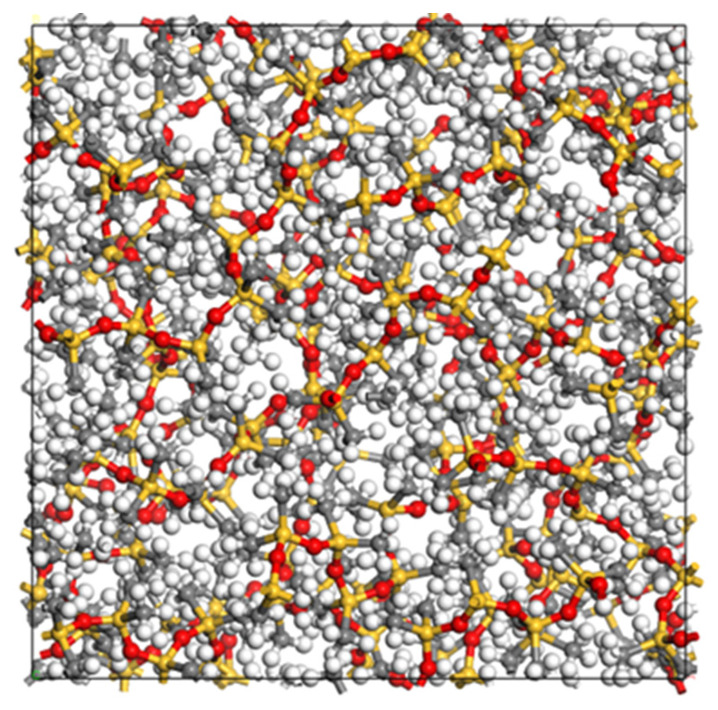
Optimized silicone rubber model.

The crosslinked model composed of four siloxane chains was further optimized using the Forcite module. The optimization process included energy minimization, geometry optimization, 200 ps relaxation in the canonical ensemble with constant particle number, volume, and temperature (NVT), relaxation in the isothermal–isobaric ensemble with constant particle number, pressure, and temperature (NPT), and subsequent relaxation in the NVT ensemble. The final optimized model is shown in Figure 4. This model contained 420 carbon atoms, 200 oxygen atoms, 204 silicon atoms, and 1248 hydrogen atoms.

Relative permittivity is an important electrical performance parameter of silicone rubber materials. To verify the rationality of the established silicone rubber model, the relative permittivity of the model was calculated using a relative permittivity script. This script calculates the relative permittivity, ε, based on the dipole moment of the model, as expressed in Equation (1).

(1) $$\varepsilon =1+\frac{1}{3Vk_{B}T\varepsilon_{0}}(\langle M^{2}\rangle -{\langle M\rangle }^{2})$$

where V is the model volume, $k_{B}$ is the Boltzmann constant, T is the temperature, $\varepsilon_{0}$ is the vacuum permittivity, *M* is the dipole moment of each frame of the model, and <> denotes the arithmetic average.

The calculated relative permittivity of the model is 6.921. This method determines the static dielectric constant based on equilibrium dipole moment fluctuations, which is comparable to the relative permittivity of practical silicone rubber measured at low frequencies. For instance, Reference [35] reported that the low-frequency relative permittivity of aged silicone rubber ranges from 4.5 to 7.5. Therefore, the silicone rubber model constructed in this study can be considered reliable. It should be noted that the established model represents an idealized crosslinked MVQ matrix without inorganic fillers, additives, residual catalysts, or other components commonly present in engineering silicone rubber materials. Therefore, the calculated relative permittivity is used only as a qualitative indicator of model rationality rather than a strict quantitative comparison with practical materials. In addition to relative permittivity, the stability of the model was further evaluated based on the structural relaxation process and the convergence of the optimized configuration. The optimized model maintained a stable crosslinked network after energy minimization and ensemble relaxation, indicating that it was suitable for subsequent reactive molecular dynamics simulations.

### 2.3. Simulation Conditions

To simulate the temperature, humidity, and electric field environment, 10 water molecules were added to the silicone rubber model shown in Figure 4 to represent the humid environment. Based on the molecular composition of the crosslinked MVQ model, the added water molecules accounted for approximately 1.18 wt% of the total system mass. Since the water absorption of common silicone rubber is usually below 1%, the amount of water introduced in this model is close to the moisture uptake level of silicone rubber under humid conditions and can be used to represent a water-vapor-intruded silicone rubber model at the molecular scale. The model was then imported into LAMMPS, and the ReaxFF command was used to apply different temperature and electric field gradients to observe the cleavage behavior of the model. Reactive molecular dynamics simulations were performed in LAMMPS using the ReaxFF force field with charge equilibration. The NVT ensemble was adopted during the simulations, and periodic boundary conditions were applied in all directions. The temperature was controlled using a thermostat during the heating, aging, and recovery stages. The external electric field was applied along a fixed direction to evaluate its effect on molecular chain scission. In this study, a uniform, positive electric field parallel to the *X*-axis was applied to the model using the fix efield command in LAMMPS. The electric field was maintained constant under each simulation condition, exerting forces on the charged atoms within the system. Product species were identified according to the bond-order information extracted from the ReaxFF trajectory, and the evolution of molecular fragments was analyzed by counting the atom numbers and species of decomposition products. The trajectory files were further used to track representative reactions, including methyl group detachment, Si-O bond cleavage, water-involved reactions, and molecular reconstruction.

For the sake of computational efficiency, it is necessary to set the temperature and electric field strength at relatively high levels. Meanwhile, when flashover occurs in electrical equipment, the material surface experiences transient high temperatures under the action of electric arcs; thus, setting a high maximum pyrolysis temperature is also of practical significance. Preliminary calculations revealed that when the maximum temperature was set to 5000 K, the statistical curve for product yield still showed an upward trend. To thoroughly investigate the electrothermal pyrolysis mechanisms of silicone rubber molecules, the maximum temperature was ultimately set to 10,000 K, and the maximum electric field strength was set to 8 V/Å.

The specific simulation conditions are listed in Table 1. To simulate the pyrolysis mechanisms of silicone rubber under various temperature and electric field conditions, all simulations were conducted independently based on the established initial model. To simulate the state of the sample after aging when returned to normal conditions, each simulation condition was divided into two stages. The first stage is the aging stage, used to simulate the degradation process of silicone rubber; the second stage is the recovery stage, during which the aging environmental factors are gradually removed to simulate the gradual recovery of the material. Each stage was simulated for 50 ps. Therefore, the elevated temperature and electric field should be regarded as accelerated simulation conditions, and the simulation results are intended to reveal possible atomistic reaction pathways and relative degradation trends under coupled temperature–humidity–electric field conditions, rather than to directly reproduce the actual long-term aging process or establish a one-to-one correspondence with real operating conditions.

## 3. Cleavage Characteristics of Silicone Rubber Under Coupled Fields

### 3.1. Pyrolysis Behavior Under Different Electric Fields

#### 3.1.1. Analysis of Siloxane Molecule Pyrolysis Mechanisms

Conditions 1, 2, and 3 were designed to investigate the pyrolysis behavior of silicone rubber under different electric fields. During the aging stage, the model was uniformly heated from 300 K to 10,000 K, while electric fields of 0 V/Å, 4 V/Å, and 8 V/Å were applied, respectively. The temperature and electric field conditions used in the simulations were much higher than those under actual service conditions. This is because the time and spatial scales of the simulations were at the picosecond level. If the simulation conditions were not sufficiently severe, the required simulation time would increase significantly, thereby greatly reducing computational efficiency.

**Figure 5:**
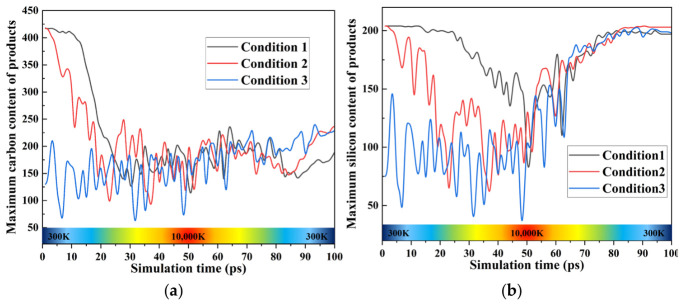
Statistics of maximum carbon and silicon content in pyrolysis products under different electric fields: (**a**) Maximum carbon content of pyrolysis products under different electric fields. (**b**) Maximum silicon content of pyrolysis products under different electric fields.

The pyrolysis products under different electric fields were statistically analyzed. Specifically, the number of carbon atoms in the fragment with the highest carbon content and the number of silicon atoms in the fragment with the highest silicon content were calculated, as shown in Figure 5. Carbon exists mainly in the form of methyl groups in silicone rubber, and these methyl groups can detach during thermal decomposition, which is a direct cause of the decrease in hydrophobicity of the sheath. Silicon exists mainly in the form of the siloxane main chain, and the fracture of Si-O bonds during aging is a direct cause of cracks on the sheath surface. During the heating process, the silicone rubber model gradually decomposes. Small molecules gradually transform into gas molecules or powdered particles and detach from the original structure, while the molecule with the largest molecular weight eventually remains in situ. Therefore, it is meaningful to analyze the carbon and silicon contents of this remaining particle.

As shown in Figure 5, the maximum carbon content of the products begins to decrease even at room temperature, indicating that methyl groups can slowly detach at room temperature. By comparing the three curves in Figure 5a, it can be seen that, in the absence of an electric field, the methyl groups in silicone rubber begin to detach rapidly at approximately 3000 K and reach a peak detachment stage at around 5500 K. Subsequently, as the temperature continues to increase from 5500 K to 10,000 K, the maximum carbon content fluctuates around 170. The reason why it does not continue to decrease may be that carbon atoms in the methyl groups further form C-C bonds and attach to the main fragment, which corresponds macroscopically to gradual carbonization of the sample. When the temperature decreases from 10,000 K to 300 K, the maximum carbon content does not change significantly but fluctuates around the previous level, indicating that the aging process associated with carbonization is irreversible for silicone rubber. When the electric field strength increases to 4 V/Å, the decrease in the maximum carbon content occurs earlier and is accelerated, while the subsequent variation trend is similar to that in Condition 1. When the electric field strength increases to 8 V/Å, the maximum carbon content decreases sharply even at room temperature, and further heating has no significant effect on the carbon content. After severe aging, the carbon contents in the three conditions show little difference and fluctuate at approximately the same level, indicating that the electric field mainly accelerates this process.

By comparing the three curves in Figure 5b, it can be seen that, in the absence of an electric field, the siloxane chains in silicone rubber begin to break rapidly at approximately 5500 K and eventually decrease to about 80. When an electric field is introduced into the aging environment, the fracture of Si-O bonds is further accelerated, and the final degree of chain scission is increased. When the electric field reaches 8 V/Å, Si-O bond fracture occurs even at room temperature. A comparison of the two figures shows that, under an electric field of 8 V/Å, both the maximum carbon content and the maximum silicon content decrease to approximately one-third of their initial values. Since the numbers of silicon and carbon atoms in the unaged siloxane chain are linearly related, it can be inferred that, under an electric field of 8 V/Å, the decrease in maximum carbon content is caused by siloxane chain scission rather than methyl group detachment. This indicates that the siloxane main chain is more susceptible to the electric field than the methyl groups.

The bond energy of Si-O is approximately 460 kJ/mol, which is higher than that of Si-C, approximately 318 kJ/mol, meaning that Si-C bonds are theoretically easier to break than Si-O bonds. Nevertheless, bond cleavage under an external electric field is not determined by bond energy alone. The Si-O bond has a stronger polar character than the Si-C bond because of the large electronegativity difference between Si and O atoms. In the simulations, the atomic partial charges are dynamically updated during the reaction process. When a constant electric field is applied, Si and O atoms with different partial charges experience electric-field-induced forces in different directions, resulting in bond polarization and bond elongation. Moreover, the siloxane backbone is a continuous chain structure. When the local orientation of a Si-O bond or a siloxane chain segment is approximately aligned with the electric-field direction, the field-induced potential difference along the chain segment can further promote backbone stretching and Si-O bond scission. In contrast, Si-C bonds are mainly located in methyl side groups, and their response to the electric field is more localized. Therefore, under a strong electric field, Si-O backbone cleavage can become more pronounced even though the Si-O bond has a higher bond energy than the Si-C bond. Unlike the maximum carbon content, the maximum silicon content gradually increases after the aging intensity is reduced and eventually recovers to its initial level. This indicates that Si-O bonds tend to reform after aging. However, the broken siloxane chains are difficult to restore to their original long-chain state, which macroscopically manifests as aggregation in silicone rubber.

This result suggests that cracks and detached particles in silicone rubber may be related to the combined effects of electric field, temperature, and non-uniform internal stress distribution generated by local chain scission, bond recombination, and molecular aggregation. Under high temperature and electric field, local Si-O bond scission occurs in the siloxane network. The broken Si-O bonds can then reconnect with surrounding siloxane fragments, resulting in local molecular aggregation and structural heterogeneity. Such reconstructed chemical bonds disturb the originally relatively balanced molecular network and may generate non-uniform internal stress distribution at the molecular scale. This non-uniform internal stress may further stretch or tear the degraded silicone rubber structure, thereby contributing to surface cracking, chalking, and particle formation. This corresponds to the aging characteristics observed in practice, such as cracking, chalking, and hardening of silicone rubber [36].

#### 3.1.2. Evolution of Decomposition Products During Pyrolysis

**Figure 6:**
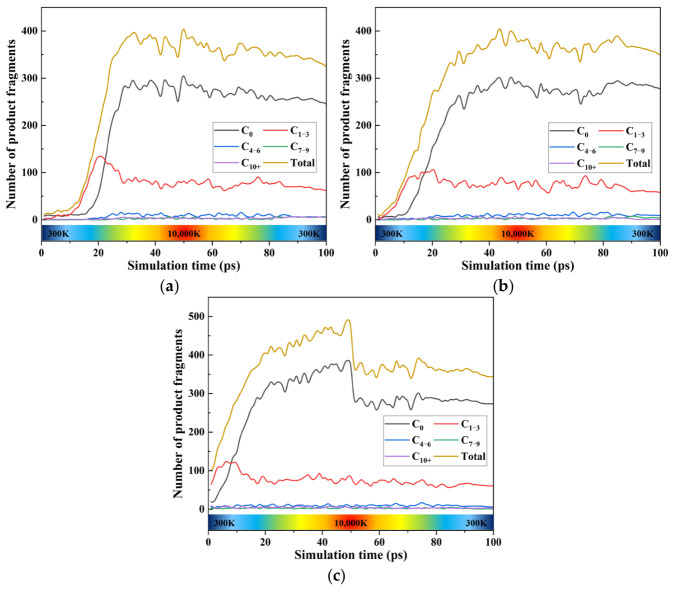
Quantity statistics of carbon-containing pyrolysis products under gradient electric field intensities: (**a**) Electric field strength 0 V/Å. (**b**) Electric field strength 4 V/Å. (**c**) Electric field strength 8 V/Å.

Further statistics were conducted on the types and quantities of products generated during the aging process to investigate the specific reaction pathways. First, the number of products with different carbon contents during aging was analyzed, including products containing no carbon, 1–3 carbon atoms, 4–6 carbon atoms, 7–9 carbon atoms, and more than 10 carbon atoms. The results are shown in Figure 6.

As shown in Figure 6, it can be observed that, during the aging stage, the total number of products for all three conditions increased rapidly with increasing temperature and eventually fluctuated around a certain value. Higher electric field strengths accelerated this process and increased the upper limit. The number of products without carbon followed a similar trend. Notably, for all three conditions, the number of products containing 1–3 carbon atoms first increased and then decreased. As their quantity declined, the number of products containing 4–6 carbon atoms increased significantly, and the number of higher-carbon products slightly increased, indicating that in the absence of an electric field, when the temperature exceeded 4500 K, small carbon-containing molecules gradually underwent dehydrogenation and deoxidation, with carbon atoms forming C-C bonds and showing signs of carbonization.

During the recovery stage, the total number of products slightly decreased, mainly due to partial recombination of siloxane small molecules as the temperature dropped, although the overall change was minor. In Condition 3 during the recovery stage, there was a sudden change in the number of carbon-free molecules, primarily because the electric field was suddenly removed, and as seen in Figure 5, Si-O bonds are highly sensitive to the electric field. This resulted in a large number of broken siloxane fragments recombining. Eventually, after cooling to room temperature, the numbers of all types of products under the three conditions were almost identical, indicating that the final siloxane cleavage outcomes were similar after severe aging.

**Figure 7:**
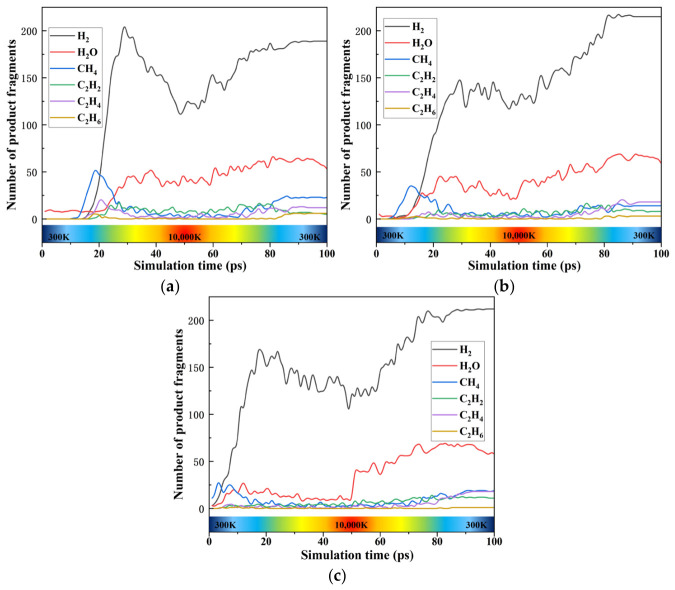
Quantity statistics of common pyrolysis products under gradient electric field intensities: (**a**) Electric field strength 0 V/Å. (**b**) Electric field strength 4 V/Å. (**c**) Electric field strength 8 V/Å.

Next, the quantities of six common products—H_2_, H_2_O, CH_4_, C_2_H_2_, C_2_H_4_, and C_2_H_6_—were statistically analyzed under the three conditions, and the results are shown in Figure 7.

As shown in Figure 7, it can be observed that in the absence of an electric field, the amount of H_2_ increased rapidly, reaching a peak at around 6000 K, then decreased as H_2_ reacted with other molecules to form higher-energy, unstable species. During the recovery stage, the H_2_ quantity gradually stabilized as the temperature decreased. The number of H_2_O molecules initially fluctuated around 10, which corresponded to the water molecules added before aging. As the temperature increased, the number of H_2_O molecules gradually increased and stabilized until the end of the recovery stage, with a slight rise. The variation in CH_4_ quantity was similar to that of the 1–3-carbon-containing products in Figure 6a, reaching a peak around 4000 K and then decreasing, while increasing slightly during the recovery stage. As CH_4_ decreased, the quantities of C_2_H_2_ and C_2_H_4_ increased, indicating that some methane molecules converted into higher-energy acetylene and ethylene during heating, along with the release of a large amount of H_2_. As the temperature further increased, the quantities of C_2_H_2_, C_2_H_4_, and C_2_H_6_ decreased, with slight increases during the recovery stage. This may be because the sudden removal of the electric field accelerated recombination between major siloxane fragments, generating additional water molecules, corresponding to the sudden drop in carbon-free products in Figure 6c. Observing the recovery stage, the final amounts of H_2_ and H_2_O increased, particularly when the 8 V/Å electric field was removed, where the number of water molecules spiked, reaching levels comparable to the other two conditions. This may be because the sudden removal of the electric field accelerated recombination between major siloxane fragments, generating additional water molecules, corresponding to the sudden drop in carbon-free products in Figure 6c.

The simulated product evolution is generally consistent with previous studies on PDMS, MVQ, and silicone rubber degradation. The generation of CH_4_, C_2_H_6_, C_2_H_4_, C_2_H_2_, and H_2_ indicates that methyl detachment, C-H bond cleavage, and subsequent radical recombination are important side-chain degradation reactions. The decrease in the maximum silicon content and the formation of carbon-free fragments indicate Si-O backbone scission, which is consistent with the reported vulnerability of siloxane networks under severe thermal or electrical stress. In addition, the formation and consumption of H_2_O, together with trajectory evidence of Si-OH formation, suggests that water molecules can participate in the reconstruction of broken siloxane chains. These results support the interpretation that silicone rubber degradation involves both side-chain reactions and backbone cleavage rather than a single decomposition route.

**Figure 8:**
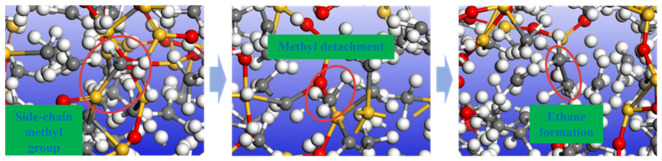
Trajectory path of ethane formation from methyl reaction.

By tracking the trajectory files of the products, certain reaction processes were observed. Figure 8 traces the reaction pathway of a methyl group under Condition 1, showing its detachment from the siloxane chain and subsequent reaction with another methyl group to form ethane, which corroborates the previous analysis.

**Figure 9:**
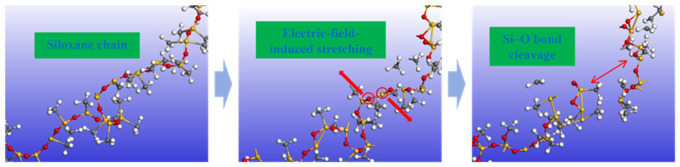
Trajectory path of siloxane chain scission under electric field.

Next, the trajectory under Condition 2 was analyzed, as shown in Figure 9, showing the first three picoseconds of the simulation when the maximum temperature reached 900 K, primarily under the effect of the electric field. Under the electric field, the silicon and oxygen atoms in the siloxane chain experienced forces in opposite directions, and eventually the Si-O bonds were broken. Due to the much higher electronegativity of oxygen compared to silicon, the electric field applied opposite forces on the two atoms, leading to bond scission under the combined action of the electric field and atomic kinetic energy.

### 3.2. Molecular Chain Scission Under Electric Field Alone

#### 3.2.1. Analysis of Siloxane Molecule Pyrolysis Mechanisms

**Figure 10:**
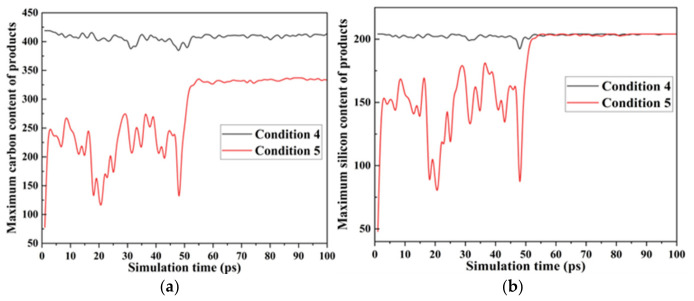
Statistics of maximum carbon and silicon content in products under different electric fields: (**a**) Maximum carbon content of products under different electric fields. (**b**) Maximum silicon content of products under different electric fields.

To further investigate the effect of the electric field on the cleavage behavior of silicone rubber, gradient electric field intensities were applied to the silicone rubber model for aging analysis, corresponding to Conditions 4 and 5. The changes in the products were then statistically analyzed. The simulation was also divided into two stages. The first 50 ps was the aging stage, during which electric fields of 4 V/Å and 8 V/Å were applied, respectively. The following 50 ps was the recovery stage, during which the electric field was removed, allowing the molecules to recover through natural reactions. The maximum carbon content and maximum silicon content of the products during the simulation were statistically analyzed, and the results are shown in Figure 10.

As shown in Figure 10, when an electric field of 4 V/Å was applied alone, no obvious cleavage of the main chain occurred, and only some methyl groups detached under the action of the electric field. Therefore, the effect of Condition 5 is mainly analyzed here. By comparing the maximum carbon content and maximum silicon content of the products under Condition 5, it can be observed that the two curves exhibit similar trends. This indicates that the variation in the maximum carbon content is mainly associated with the cleavage of Si–O bonds, accompanied by the detachment of some methyl groups. Overall, the quantities fluctuate within a certain range, suggesting that the siloxane main chain continuously undergoes cleavage and recombination under the combined effects of thermal motion and the electric field. In the recovery stage, similar to the pyrolysis stage, the siloxane chains broken under the electric field tend to reconnect. However, some detached methyl groups cannot recover and are permanently released as gaseous products such as methane.

#### 3.2.2. Evolution of Decomposition Products Under Electric Fields Alone

**Figure 11:**
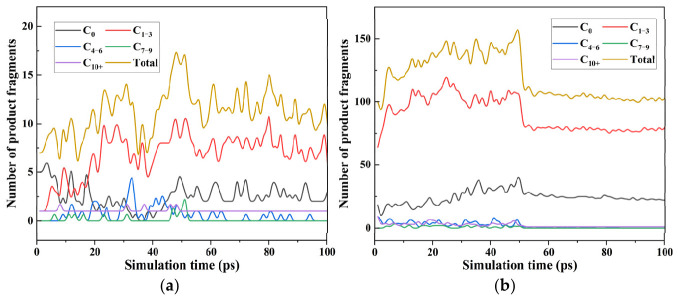
Quantity statistics of different carbon-containing products under gradient electric field intensities: (**a**) Electric field strength of 4 V/Å. (**b**) Electric field strength of 8 V/Å.

The number of carbon-containing products under gradient electric fields was then statistically analyzed. Products were similarly classified into carbon-free products and products containing 1–3 carbon atoms, 4–6 carbon atoms, 7–9 carbon atoms, and more than 10 carbon atoms. The results are shown in Figure 11.

As shown in Figure 11, when the electric field strength was 4 V/Å, the cleavage products were mainly a small number of carbon-free molecules and some molecules containing 1–3 carbon atoms. Notably, when the simulation entered the recovery stage, the quantity trend of the main carbon-containing products showed almost no change and maintained a fluctuation pattern similar to that in the aging stage. This indicates that, under this electric field strength, the electric field only promoted the decomposition of the sample; therefore, after the electric field was removed, the detached molecules did not recover.

When the electric field strength was 8 V/Å, the cleavage products were also mainly carbon-free molecules and some molecules containing 1–3 carbon atoms. After the electric field was removed, the quantities of various molecules decreased rapidly and eventually stabilized at a fixed level. This indicates that, under this electric field strength, the electric field played a dominant role in product decomposition. Therefore, after the electric field was removed, the potential energy of the system decreased rapidly, causing some small molecules to recombine with macromolecules.

**Figure 12:**
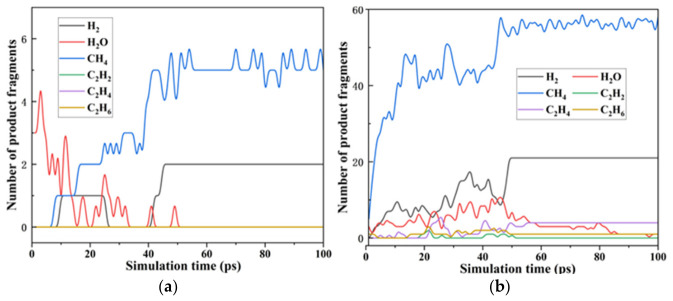
Quantity statistics of common products under gradient electric field intensities: (**a**) Electric field strength of 4 V/Å. (**b**) Electric field strength of 8 V/Å.

The quantities of six common products, namely H_2_, H_2_O, CH_4_, C_2_H_2_, C_2_H_4_, and C_2_H_6_, were also statistically analyzed under these conditions. The results are shown in Figure 12.

As shown in Figure 12, when the electric field strength was 4 V/Å, the amount of methane increased continuously, while the amount of water molecules decreased continuously until disappearing. After the electric field was removed, the quantities of both species showed no obvious change. Hydrogen was briefly generated and then disappeared during the aging stage. After the electric field was removed, hydrogen began to form again and remained at a stable quantity. This may be because hydrogen was converted into higher-energy species during aging and then naturally decomposed back into hydrogen after the electric field disappeared. When the electric field strength was 8 V/Å, methane showed a variation trend similar to that under 4 V/Å. The amounts of hydrogen and ethylene increased and fluctuated, and after the electric field was removed, they also increased and stabilized at certain values. The number of water molecules gradually increased under the electric field, but after the aging stage ended, it rapidly decreased and eventually disappeared. Since the quantities of acetylene and ethane changed only slightly, they are not discussed in detail here.

#### 3.2.3. Effect of Water Molecules on the Degradation Process of Silicone Rubber

**Figure 13:**
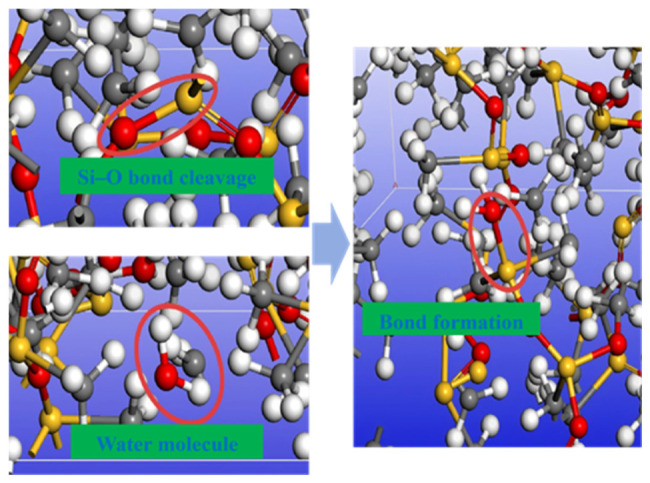
Trajectory path of water molecule involved in reaction.

It is worth noting that ten water molecules were included in the model during construction. During the electro-thermal aging process, the reactions tend to favor the generation of water molecules. These water molecules participate in numerous reactions and intermediate processes, making the overall reaction pathways difficult to quantify statistically. However, after independent electric-field aging, these water molecules disappeared. Therefore, the reactions involving water molecules during this process need to be further analyzed based on the corresponding trajectory files. Figure 13 shows the trajectory file under Condition 4, tracing the process in which a silicon atom evolves from being part of an intact Si-O bond to bond cleavage and subsequent reaction. It can be observed that, under the action of the electric field, the Si-O bond breaks and then reacts with a water molecule to form a hydroxyl group. This bonding remains stable even after the aging process ends. Therefore, it can be considered that moisture ingress accelerates the degradation of silicone rubber. Water molecules occupy the original Si-O bond structure, causing long chains to transform into short chains, and this aging process is irreversible. At the macroscopic level, this leads to particle formation in silicone rubber and significant structural damage.

**Figure 14:**
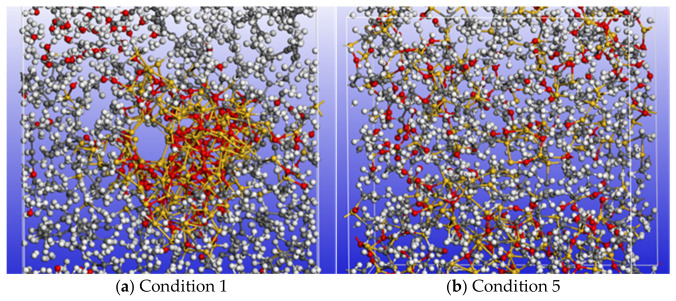
Atomic trajectories after the completion of two aging recovery stages.

The morphologies of the thermally aged sample and the electric-field-aged sample at the end of the recovery stage were observed, as shown in Figure 14. Figure 14a shows the final trajectory of Condition 1 during the recovery stage, while Figure 14b shows the final trajectory of Condition 5 during the recovery stage.

As shown in Figure 14a, after thermal aging, a large number of methyl groups in the siloxane structure detach, generating small gaseous molecules such as methane and ethane. Meanwhile, extensive cleavage and reconstruction occur in the siloxane chains. Detached oxygen atoms combine with hydrogen atoms to form a small amount of water molecules or alcohol-like molecules. The reconstructed siloxane structures exhibit obvious aggregation.

As shown in Figure 14b, simple reconstruction occurs in the model under the action of the electric field. Although some Si-O bonds break under the electric field, after the electric field is removed, the short silicone rubber chains tend to reconnect through oxygen atoms, carbon atoms, or direct Si-Si bonding. Therefore, the maximum silicon content of the products shown in Figure 5, Figure 6, Figure 7, Figure 8, Figure 9, Figure 10 and Figure 11 recovers to its initial value after the aging stage.

Based on the above observations, it can be concluded that the effect of pure thermal aging on siloxane molecules mainly consists of two stages. In the first stage, methyl groups detach from the siloxane chains. In the second stage, extensive Si-O bond cleavage occurs. The first stage exists continuously during daily aging and is mainly caused by local heating of the bushing or temperature rise induced by sunlight exposure. The second stage mainly occurs when an arc is generated. The temperature during arcing is usually 3000–4000 °C, which can cause rapid local thermal aging of silicone rubber. Electric-field aging causes local fracture of silicone rubber, tending to transform siloxane chains from long-chain structures into short-chain structures. At the same time, it also causes partial methyl group detachment, indicating that these two reactions occur simultaneously.

The microscopic reactions tracked during the simulations also exhibit a strong correlation with the macroscopic aging characteristics of practical silicone rubber. For instance, the breaking and recombination of Si-O bonds cause uneven internal stress, leading to cracks and pulverization during aging. The further propagation of these cracks allows moisture intrusion, which ultimately causes insulation failure of the sheath and triggers breakdown discharges. Additionally, the detachment of methyl groups results in the loss of hydrophobicity. In rainy environments, this leads to water droplet adhesion on the surface and electric field distortion, eventually causing the loss of external insulation and flashover arcs. Furthermore, the microscopic pyrolysis processes—specifically the scission of Si-O bonds and the detachment of methyl groups—are consistent with the decreased characteristic peaks of Si-O and Si-C bonds observed in FTIR tests of aged silicone rubber. The high-temperature reactions of siloxanes also correspond, to a certain extent, to the reactions occurring during flashover arcs in silicone rubber.

### 3.3. Microscopic Degradation Mechanism of Silicone Rubber

Based on the product evolution in Figure 5, Figure 6, Figure 7, Figure 8, Figure 9, Figure 10, Figure 11 and Figure 12 and the trajectory analysis in Figure 8, Figure 9, and Figure 13, the degradation of silicone rubber can be interpreted as a competition between bond cleavage and bond reconstruction.

#### 3.3.1. Cleavage Reactions of Siloxane

**Figure 15:**
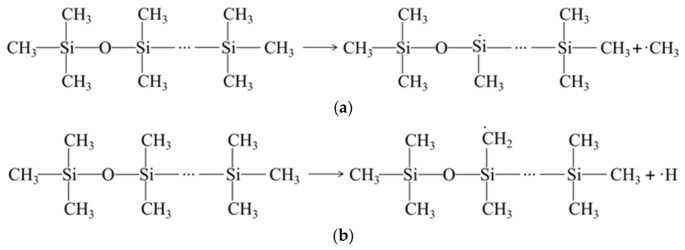
Cleavage of siloxane side chains at room temperature. (**a**) methyl detachment. (**b**) methyl dehydrogenation.

Siloxane cleavage can occur under conditions ranging from room temperature to high temperature, as well as under an electric field, although the specific reaction processes differ. The pyrolysis of siloxane molecules can be divided into two parts: the scission of side-chain methyl groups and the cleavage of main-chain Si-O bonds. Furthermore, the conditions required for these two types of reactions are different. At room temperature, siloxane cleavage is mainly characterized by methyl group detachment and methyl group dehydrogenation. These reactions may occur under ultraviolet radiation or oxidative environments, and can be accelerated by increasing temperature or enhanced electric field strength. The specific reaction process is shown in Figure 15.

**Figure 16:**
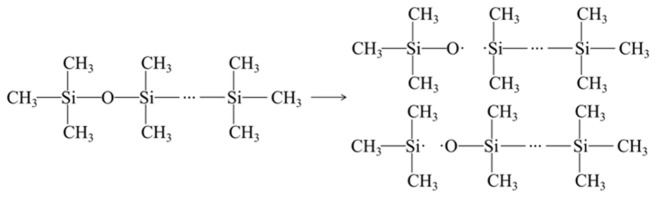
Cleavage of siloxane main chain at room temperature.

When silicone rubber is exposed to external force, intense ultraviolet radiation, acidic or alkaline environments, or strong electric fields, Si-O bonds can break, causing long siloxane chains to transform into shorter chains. The specific reaction process is shown in Figure 16.

**Figure 17:**
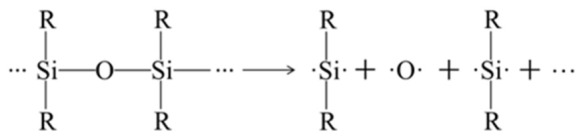
Massive cleavage of silicon-oxygen bonds at high temperature.

When surface arc discharge occurs on the outer surface of the bushing, the silicone rubber surface is subjected to instantaneous high temperature and a strong electric field. Under such high-temperature conditions, Si-O bonds rapidly cleave, leading to complete fracture of the siloxane chains and the formation of a large number of free radicals. The reaction process is shown in Figure 17. Since the aging conditions are severe in this case, the side-chain methyl groups may have already changed; therefore, R is used to represent the side-chain groups.

#### 3.3.2. Bonding Reactions After Cleavage

The bonding reactions after siloxane cleavage can also be divided into two cases: reactions at room temperature and reactions at high temperature.

**Figure 18:**
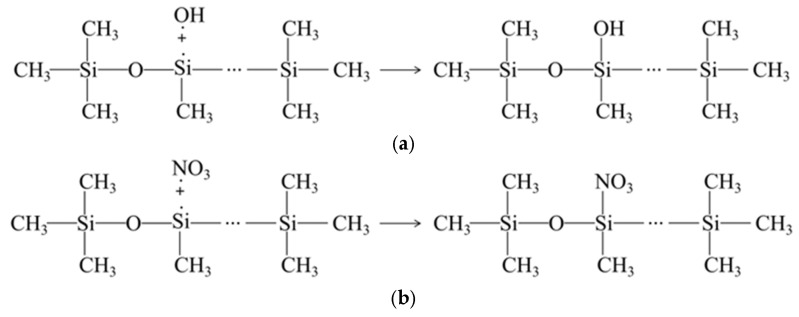

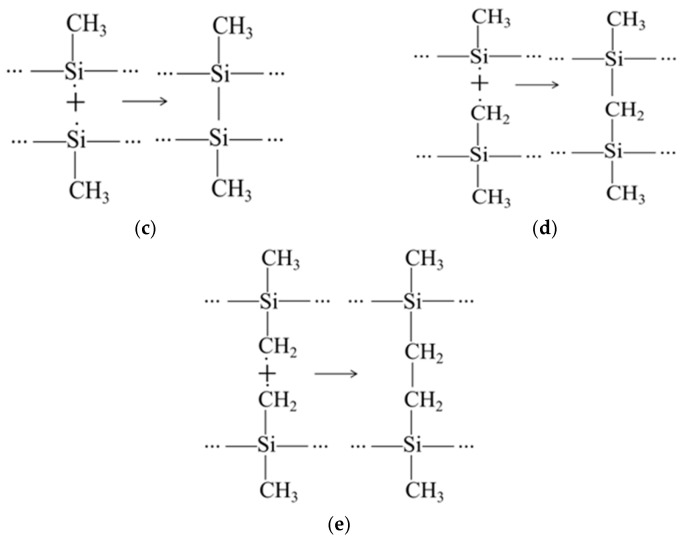
Bonding reaction of side chains at room temperature. (**a**) Si–OH bond formation. (**b**) Si–NO_3_ bond formation. (**c**) Si–Si bond formation. (**d**) Si–C bond formation. (**e**) C–C bond formation.

At room temperature, due to methyl group detachment or dehydrogenation, an unpaired electron remains at the original position. This site often reacts and connects with hydroxyl groups, nitrate groups in the air, or nearby siloxane structures, resulting in crosslinking between siloxane molecules and the formation of a denser crosslinked network. The corresponding side-chain bonding reactions are shown in Figure 18.

**Figure 19:**
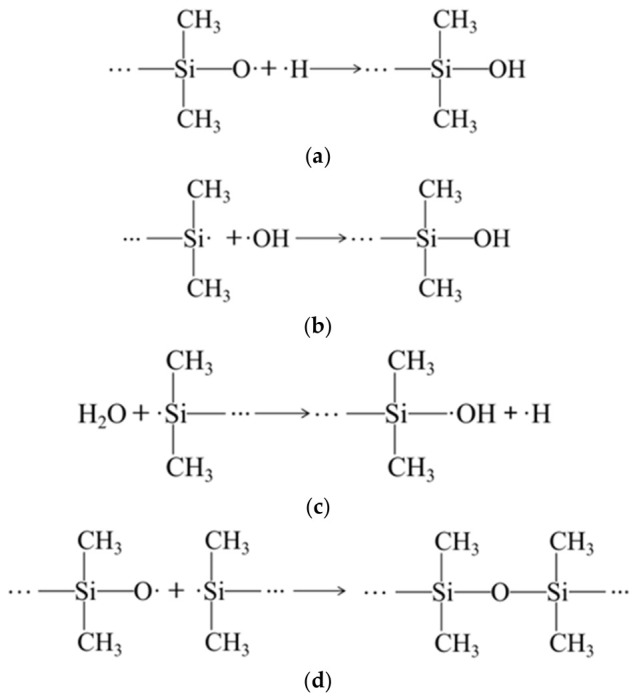
Bonding reaction of main chain at room temperature. (**a**) hydroxyl group formation. (**b**) Si–OH bond formation. (**c**) water-involved bonding reaction. (**d**) Si–O bond formation.

At room temperature, the main chain may break under strong electric fields or other environmental stresses. After cleavage, the exposed silicon atoms can react with water molecules, hydroxyl groups, nitrate groups, and other species, while oxygen atoms can reconnect with hydrogen ions or other exposed silicon atoms produced by chain cleavage. The specific reactions are shown in Figure 19.

**Figure 20:**
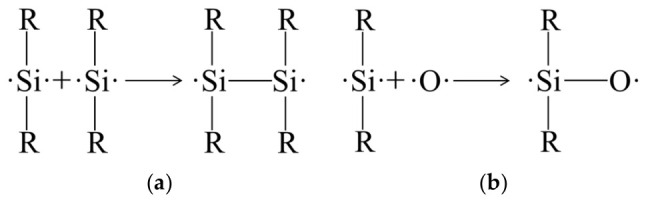
Bonding reaction of main chain at high temperature. (**a**) Si–Si bond formation. (**b**) Si–O bond formation.

When arc discharge occurs on the silicone rubber surface, local Si-O bonds undergo extensive cleavage at high temperature and then reform. Bonding may occur between cleaved silicon atoms, or between silicon atoms and oxygen atoms. In this process, the original structure is completely destroyed, and aggregated structures containing silicon and oxygen atoms are regenerated, as shown in Figure 20.

**Figure 21:**
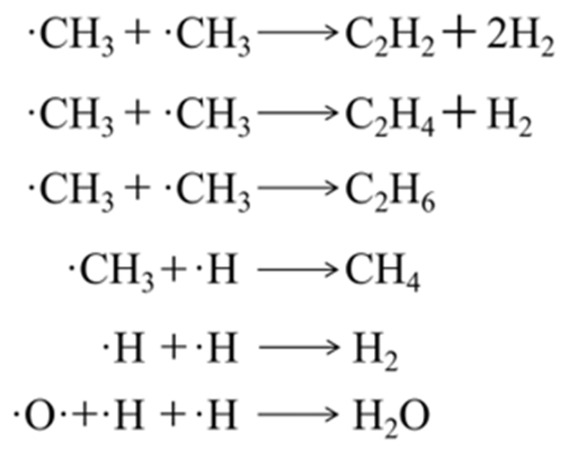
Bonding reaction of radicals at high temperature.

Meanwhile, the decomposed free radicals react with one another, generating small molecules such as methane, ethane, ethylene, acetylene, hydrogen, and water molecules. These small molecules may also undergo oxidation reactions at high temperature and be consumed. The bonding reactions between these radicals are shown in Figure 21.

## 4. Discussion

In this study, a cross-linked silicone rubber model was established, and LAMMPS was employed to simulate the pyrolysis of the model under high temperature, strong electric fields, and moisture. By analyzing the degradation parameters and reaction products of the model, we obtained the pyrolysis mechanisms and atomic trajectories of silicone rubber under different electrothermal environments. Although some conclusions have been drawn, this study still has certain limitations.

(1) Considering simulation efficiency and the need to explore the complete pyrolysis mechanism of silicone rubber, the maximum temperature was set to 10,000 K in this study. Although local temperatures can reach thousands of degrees during flashover events in power equipment—providing some practical relevance for the 10,000 K setting—the appropriateness of this temperature for investigating the pyrolysis mechanisms requires further validation. Future studies could employ simulations with varying maximum temperatures to investigate the degradation behavior of silicone rubber after cooling under different peak aging temperatures.
(2) During the electrothermal aging simulation in this study, a large amount of water molecules was generated, leading to an excessive number of transition reactions involving water. This made statistical analysis highly challenging. Consequently, the investigation into water-involved reactions has certain limitations. Future studies could employ milder reaction conditions to track the specific reaction pathways of water molecules.
(3) Due to limitations in simulation capabilities and computational power, the investigation into the pyrolysis mechanisms and pathways of silicone rubber is not sufficiently in-depth. Many reaction pathways occurring during the simulation have not been statistically captured. Future work requires further refinement of the statistical scripts to conduct computational studies on specific reactions at smaller scales and with higher precision.

## 5. Conclusions

This study used ReaxFF reactive molecular dynamics simulations to investigate the degradation behavior of methyl vinyl silicone rubber under coupled temperature, humidity, and electric field conditions. A crosslinked molecular model was established and verified through structural optimization and relative permittivity calculation, providing a basis for subsequent aging simulations. By analyzing product evolution and atomic trajectories, the molecular chain scission, radical reactions, and structural reconstruction processes during silicone rubber aging were clarified.

(1) The results show that high temperature mainly promotes methyl group detachment, C-H bond cleavage, and Si-O backbone scission. As the temperature increases, the maximum carbon and silicon contents of decomposition products decrease, indicating progressive side-chain degradation and siloxane main-chain cleavage. These structural changes are largely irreversible during the recovery stage.
(2) The electric field further accelerates molecular decomposition and has a pronounced effect on the siloxane backbone. A weak electric field mainly induces limited methyl detachment, whereas a strong electric field can cause significant Si-O main-chain cleavage even at room temperature. This indicates that electric-field-induced stretching and local structural distortion play important roles in silicone rubber degradation.
(3) Water molecules participate in the aging process after Si-O bond cleavage. Exposed silicon atoms can react with water molecules to form hydroxyl-containing structures, which prevents the original siloxane chains from fully recovering. This water-assisted reaction shortens the molecular chains and aggravates irreversible structural damage.

Overall, silicone rubber degradation under coupled temperature–humidity–electric field conditions involves side-chain methyl detachment, Si-O main-chain cleavage, radical recombination, water-assisted hydroxyl formation, and local siloxane chain reconstruction. These reactions lead to reduced hydrophobic groups, chain shortening, local aggregation, and structural heterogeneity, providing a molecular-level explanation for macroscopic aging phenomena such as hydrophobicity loss, cracking, chalking, and particle shedding. The findings provide theoretical support for understanding the aging mechanism and condition evaluation of silicone rubber used in composite external insulation materials.

## Figures and Tables

**Table 1:** Simulation conditions for reaction kinetics.

Condition	Temperature (K)	Electric Field Strength (V/Å)
1	300–10,000 10,000–300	00
2	300–10,000 10,000–300	40
3	300–10,000 10,000–300	80
4	300	40
5	300	80

## Data Availability

The original contributions presented in this study are included in the article. Further inquiries can be directed to the corresponding author.
